# Social Network Communications in Chilean Older Adults

**DOI:** 10.3390/ijerph17176078

**Published:** 2020-08-21

**Authors:** Francisco Javier Rondán-Cataluña, Patricio E. Ramírez-Correa, Jorge Arenas-Gaitán, Muriel Ramírez-Santana, Elizabeth E. Grandón, Jorge Alfaro-Pérez

**Affiliations:** 1 Department Business Management and Marketing, University of Seville, 41004 Sevilla, Spain; rondan@us.es (F.J.R.-C.); jarenas@us.es (J.A.-G.); 2 School of Engineering, Universidad Católica del Norte, Coquimbo 1780000, Chile; jalfaro@ucn.cl; 3 Department of Public Health, Faculty of Medicine, Universidad Católica del Norte, Coquimbo 1780000, Chile; mramirezs@ucn.cl; 4 Department of Information Systems, Universidad del Bío-Bío, Concepción 4030000, Chile; egrandon@ubiobio.cl

**Keywords:** social networking sites, older adults, decision trees, segmentation, Chile

## Abstract

The growth of older adults in new regions poses challenges for public health. We know that these seniors live increasingly alone, and this impairs their health and general wellbeing. Studies suggest that social networking sites (SNS) can reduce isolation, improve social participation, and increase autonomy. However, there is a lack of knowledge about the characteristics of older adult users of SNS in these new territories. Without this information, it is not possible to improve the adoption of SNS in this population. Based on decision trees, this study analyzes how the elderly users of various SNS in Chile are like. For this purpose, a segmentation of the different groups of elderly users of social networks was constructed, and the most discriminating variables concerning the use of these applications were classified. The results highlight the existence of considerable differences between the various social networks analyzed in their use and characterization. Educational level is the most discriminating variable, and gender influences the types of SNS use. In general, it is observed that the higher the educational level, the more the different social networking sites are used.

## 1. Introduction

The behavior of older adults is increasingly important in our society, but the knowledge we have about it is limited. Globally, the group of people over 65 is growing faster than any other age segment. United Nations estimates indicate that by 2050 the current proportion of over 65 will double, reaching 16% of the world’s total population. Additionally, demographic data indicates that elderly adults live increasingly alone and without the company of their close relatives [1]. This situation of isolation causes depression problems and, consequently, an impairment in the general health and wellbeing of this age group. All these trends pose new challenges for public health, and especially in regions where this fact is recent.

For this reason, and as it is a global phenomenon, various digital technologies have been launched on the market that aim to obtain health benefits. Some of them have focused on improving safety and optimizing the management of some medical pathologies [2,3]. For example, multiple applications provide notification in real-time when an elder adult suffers a fall; vital monitor signs, blood glucose, and oxygen levels; send reminders for medical appointments or vaccinations [2,4,5]. In particular, in the case of social networking sites (SNS), the medical literature emphasizes their effectiveness in improving the quality of life of elderly adults [6]. Previous research has shown that the use of SNS by elderly adults reduces isolation, improves social participation, and increases autonomy [7,8,9]. From the health point of view, those elderly adults who use SNS tend to have fewer chronic pathologies and fewer depressive symptoms; while increasing their perception of wellbeing [8,9]. For example, elderly adults relate the reduction in their levels of loneliness to their participation in SNS in Slovenia [10]. Furthermore, the use of SNS by this aged population in China improves the perception of their satisfaction with life, by decreasing isolation and increasing autonomy; these relationships being influenced by social support [8].

However, even though the phenomenon of adoption of information and communication technologies (ICT) by elderly adults has been studied, and beyond stereotypical assumptions about older adults, the lack of appropriate theoretical frameworks is a fact [11]. Moreover, there is a lack of scientific research that contributes to segmenting more aged based on their characteristics and perceptions of technology. In this sense, and recognizing the benefits of using the SNS by older people [7,8,9], the need to explain the phenomenon in question is clear.

Although these facts position aging and the use of SNS as a social phenomenon of great interest to the world, it is even more relevant in Chile. According to the last Chilean CENSUS, 16.2% of Chileans are over 60 years old, and although that is the age when retirement begins in the country (60 years for women and 65 for men), 28% of them are still working. This reality is related to the structural transformation of the Chilean population pyramid, which has gone from a triangular shape in 1975 to its current rectangular shape [12]. Thus, the aging index (number of people over the age of 60 for every 100 under the age of 15), went from 35.4 to 86 between 1990 and 2015 [13]. This value is close to the average for the countries of the Organization for Economic Cooperation and Development (OECD), but much higher than the 39.6 average for Latin America [14]. In this context, there is a lack of studies that contribute to understanding elder adults and their perceptions of technology, which would support the generation of differentiated public policies that help manage the impact that the sustained increase of this age group in Chile is causing.

From a global perspective, two concepts are worth mentioning, the generational effect and the elderly digital divide. First, the generational effect refers to behavioral outcomes resulting from the synergy between age and technology use [15]. Mainly, the generational differences in using technology emerge due to physical and cognitive changes [16]. In technological context, these generational differences are associated with increasing selectivity of SNS’s partners with age [17], building and maintaining social capital [18], and decreasing the importance of past privacy issues as age increases [19], among others. Second, the digital divide between elderly adults is one of the aspects that most worries western society. Being ostracized from the advances that ICT can bring to each person’s life is a mistake that cannot be made. Even people who have had previous contacts in their professional lives with computers and who should perform well with new technologies tend to have difficulties managing them. The issue of knowing how older adults are regarding the use of new technologies is essential to address, especially in a South American country like Chile, where there are very few studies on the subject [20,21].

One of the main advances of ICT in the consumer context is in the use of SNS. Every day more people worldwide live connected to the main SNS (Facebook, Twitter, YouTube, Instagram), and the number of its users increases dramatically. At the end of 2019, Facebook had more than 2.5 billion users, Twitter 330 million, YouTube 1.5 billion, and Instagram 800 million [22]. This practically free and universal communication means can be crucial for the group of elderly adults, in which loneliness and isolation trigger physical and mental health problems. These social networks could allow these elderly groups to connect with old friends, who may live far away or have difficulties getting around, and with family members who have little time to visit and learn about their surroundings quickly. Social networks are a means of communication in which elders can be accessed in a direct and personalized way and become a form of healthy entertainment, especially for people who have considerable free time. For all these reasons, and given that these tools have different peculiarities, it is essential to know which variables influence the use of various SNS by older adults. Furthermore, being able to classify different segments of users or non-users within this age group is key to understanding the phenomenon and being able to take appropriate actions to promote the use of these new technological tools in our elderly.

Particularly, in this work, we analyze what the elderly users of social networks in Chile are like. What are the most used SNS? What are the differences in the use of SNS by elders? Are there diverse segments of users among the elderly? What variables are more relevant to segment? Giving answers to these research questions are the objective of this work. This knowledge can be used to develop strategies so that older adults are better communicated and enjoy greater physical and psychological wellbeing.

The rest of the paper is organized as follows: In the next section, we review the literature associated with ICT and elders. Then, we outline the research methodology and, subsequently, we present the main results. We then discuss the empirical findings related to each of the SNS considered in the research. Finally, we state the contributions and limitations of the study, future research opportunities, and provide concluding remarks.

## 2. Literature Review

### 2.1. ICT Phenomenon in Elder People

The World Health Organization [23] has defined active aging as “the process of optimizing opportunities for health, participation, and security to enhance the quality of life as people age.” The term “active” suggests “continued participation in social, economic, cultural, spiritual, and civic issues, not just the ability to be physically active.” In this sense, ICT and SNS appear as an essential means to promote this objective [24]. More recently, the World Health Organization [25] set the top 10 priorities for older people to fit into the 2030 Agenda for Sustainable Development. These priorities include strengthening global networks to make cities and communities friendlier to the elderly and reducing inequalities. In this new context, ICTs and SNS reappear as crucial tools for this purpose.

Describing the relationship between older people and technologies is not easy. If we start from a global perspective, the elderly offer lower penetration rates than the rest of the population in technologies such as the Internet, smartphones, and SNS [26]. A possible explanation of this fact is how this segment deals with technology. The youngest segment sees the Internet and social networks as a new virtual world where they can have fun, express, and develop. For the elderly, this virtual environment is not a fun place but a simple communication tool [27]. It is difficult to think of the elder segment as highly innovative. From the acceptance of technologies [28], they are probably more inclined to use technologies well established in the market. That is, we will find more seniors among the group of late adopters than among the pioneers. The reasons that explain these differences are multiple [26]: lack of interest, probably because they do not need to use these technologies for their daily lives; lack of skills to use the Internet and social networks; and the lower need to search for information than the rest of the population. Therefore, older people probably use virtual services less when they are available through other more “traditional” channels. All these elements push for the persistence of a stereotype of elder people seen by society as clumsy and far from technology [24].

However, this stereotypical image hides a much larger reality. Possibly the elderly is one of the segments with the most significant behavior heterogeneity [29]. The development of studies on heterogeneity among the elderly, through segmentation, question the current validity of this stereotype [30].

### 2.2. Elder Segments in the Technological Scope

Previous research that analyzed the heterogeneity of the elderly concerning ICTs were based mainly on demographic variables, such as age and gender. For example, Mathur, Sherman, & Schiffman [31] identified an elder segment called new age, characterized by the fact that they perceive themselves as younger, and their behavior is similar to that of younger people. Fox [32] also differentiates between the so-called silver tsunami, with ages up to 64 years old and inactive Internet users aged 80 and over. Subsequent research included psychographic variables, which have a higher explanatory power of the elderly´s behavior on the Internet [24]. For example, Sudbury and Simcock [33] identify the segment of positive pioneers as those older people who carry out more activities, have more social relationships, are more present on the Internet, and care, especially, what others think of them. Hong et al. [34] consider cognitive age as the factor that allows the elderly to be segmented regarding the acceptance of information technologies. All these investigations were carried out with a-priori segmentation technique, where the segmentation criteria, whether demographic or psychographic, were to be used first.

The latest research provides the use of a-posteriori segmentation techniques. These techniques allow segmenting without making a presumption of a criterion that perfectly explains the behavior of consumers. Thus, Villarejo-Ramos et al., [26] develop a segmentation of latent classes of adults over 55 based on the use they make of online services. Specifically, they take a utilitarian service, such as electronic banking, and a hedonic service, such as the use of SNS. They help to explain the segments of demographic variables (age, educational level, and gender) and psychographic variables (daring, perceived physical conditions, self-confidence, and technological anxiety). As a result, they obtained five segments. The first cluster, called e-elderly, characterized by very active behavior in both SNS and electronic banking, is the most significant segment and is far removed from the previously described stereotype. The second segment, called e-user, has great use of electronic banking but low use of SNS. This segment is mainly made up of men who give high importance to tools of utilitarian nature. The third segment, called hooked by networks, have a high use of SNS, and shallow use of electronic banking. This segment is mostly made up of women who attach great importance to hedonistic motives. They use the SNS to learn about fashion, culture, and tourism, and offer e-WOM and comments to their contacts. The fourth segment, called surfing with the family, uses the SNS, although it does not have a formal account in it. This segment is mainly made up of older women who use SNS to keep in touch with their relatives. Finally, the last segment, called fearful of technology, is about 25% of the sample. This segment is the only one that perfectly fits the previously described stereotype of those far from technology.

In conclusion, although some works have addressed the segmentation of the elderly towards ICTs and SNS, it is still a topic that has been scarcely investigated. Most research has focused on the United States, European countries, and Southeast Asia. There is very little research focus on older people from Latin American countries. Besides, concerning the statistical techniques used, we have not found works with a predictive character as offered by data mining techniques, and particularly based on the CHAID method for decision trees.

## 3. Materials and Methods

### 3.1. Data

A survey was conducted through a face to face encounter between the person administering the questionnaire and the respondent. In total, 384 older adults were surveyed, who reside in two regions separated by almost 1000 km in Chile (Coquimbo-La Serena conurbation in the Coquimbo region and the city of Concepción in the Biobío region). The regions of Coquimbo (northern Chile) and Biobío (southern Chile) were considered, since they present a significant proportion of the population over 60 years of age. According to data from the National Statistics Institute (INE), the percentage of elderly adults in the Coquimbo region is 16.7%, while in the Biobío region is 17.3%.

For data collection, a stratified sampling method was carried out. The most updated population census in the country (XIX INE National Population Census) for the Coquimbo and Biobío regions was used as a sampling frame. The results of the IX Internet Access and Use survey of the Undersecretary of Telecommunications of Chile were used to determine the proportion of Internet users by gender and age range, whose information constitutes the most up-to-date study available to the country at the time of conducting the survey. Interviewees who report that they have used the Internet for three months or less were considered users. After filtering the data and calculating the proportions, a total population of 156,401 older adults was obtained. We proposed a stratified procedure by simple affixing to determine the sample size, considering for this a maximum allowed error of 5%. This procedure is described below. First, the total sample size is calculated using the finite sample random sampling formula indicated in Equation (1):

(1) $$n=\frac{Nz_{\alpha /2}^{2}s^{2}}{e^{2}\left(N-1\right)+z_{\alpha /2}^{2}s^{2}}$$

where *n* is the size of the global sample, *N* is the size of the population defined in the sampling frame, *Z*_*α*⁄2_ is the critical value of a standardized normal random variable for a confidence level of 1 − *α*, *s*^2^ is the variance of the responses, and *e* is the maximum error allowed for the estimators of interest. Since the indicators of interest are proportions (measurement scales), for the calculation of (1) the maximum variance of a Bernoulli process is adopted, which is equal to 0.25 (the variance of a Bernoulli process is equal to p (1 − p), where p corresponds to the probability of success. Therefore, the maximum value of such variance is reached with p = 0.5). For the global sample, a confidence level of 95% is also adopted. Thus, the calculation of the global sample is reduced to the following:

(2) $$n=\frac{\left(220,970\right){\left(1.96\right)}^{2}\left(0.25\right)}{0.05^{2}\left(220,969\right)+{\left(1.96\right)}^{2}\left(0.25\right)}\;≃384$$

As a second step, the global sample is distributed in the strata by simple affixation. A sample of 384 valid questionnaires was obtained, and the sampling error is lower than 5%. Data collection was carried out in places of affluence of older adults who use SNS, such as senior and medical centers, universities that offer elderly adult programs, among others. Fifty-six point five (56.5) percent of the sample are women, and the average age is 68.7, ranging from 60 to 92 years old.

### 3.2. Statistical Tool

The decision tree technique is used in this study. This technique creates a tree-based classification model, which classifies cases or predicts values of a dependent variable based on values of independent variables. This technique provides validation instruments for exploratory and confirmatory classification analysis. The procedure can be used for different tasks:

- Segmentation: determine the people who could be members of a specific cluster.
- Stratification: assign cases to a specific category.
- Prediction: create guidelines and use them to forecast future events.
- Data reduction and variable classification: select a subset of predictors from a large set of variables to use in developing a parametric model.
- Interaction identification: identify relationships that belong only to specific subsets for later use in a parametric model.
- Merging of categories and discretization of continuous variables: recode the continuous variables and the categories of the group’s predictors with minimal loss of information.

In this case, we seek two of these tasks. First, to make a segmentation to analyze the different groups of elders who use SNS. Second, to classify the variables that discriminate the use of SNS by elders to make other elaborate parametric analyzes in the future.

The dependent variables used are dichotomous, where respondents were asked if they use Facebook, Twitter, Instagram, WhatsApp, and YouTube. Five trees were made, one for these dependent variables. The independent variables used are socioeconomic class, the number of people who live with the elder, marital status, educational level, gender, whether the elder lives with his/her partner and if work or not, age, and the use of wearable devices (smartwatch or bracelet). All of these sociodemographic variables are widely used to segment populations in previous studies [24,26,29].

The growth method used to expand the tree is CHAID (CHi-square Automatic Interaction Detection) [35]. At each step, CHAID chooses the independent variable with the most robust interaction with the dependent variable, based on a Chi-square test. The categories of each predictor are united if they are not significantly different concerning the dependent variable. The analysis begins with a root node where all the subjects are. Branches appear in the tree with child nodes that contain subsets of subjects. In the terminal nodes, the remaining groups are the most homogeneous possible for the dependent variable. These types of trees are non-parametric procedures, so there is no need to make any assumptions about the data [36].

The maximum number of levels obtained was three, and the minimum number of cases for the father and child nodes were 15 and five, respectively. Cross-validation was also applied to assess the goodness of fit of the tree structure [37]. The sample is divided into ten subsamples or folds. Subsequently, tree models are generated, including data from nine of the ten subsamples. The first tree is based on all cases except those corresponding to the first fold of the sample; the second tree is based on all cases except those of the second fold of the sample and so on. For each tree, the risk of misclassification is calculated by applying the tree to the subsample that was excluded when it was generated. The risk estimation through cross-validation for the final tree is calculated as the average of all the trees´ risks. The statistical software utilized was SPSS 23.0 (SPSS, Inc., Chicago, IL, USA).

### 3.3. Ethical Considerations

All the subjects gave their notified consent before they participated in the study. The research was conducted following the Declaration of Helsinki. The protocol was approved by the Ethics Committee of the Faculty of Medicine of the Universidad Católica del Norte (Resolution No. 14 of 6 August 2019), guaranteeing the safeguard of the ethical principles for research declared by the committee.

## 4. Results

We present the five analyzes made for the five SNS used in the study (YouTube, Facebook, Instagram, Twitter, and WhatsApp). Some of these SNS have many users among those surveyed and others not.

### 4.1. Characterization of Elder Chileans YouTube Users

**Figure 1:**
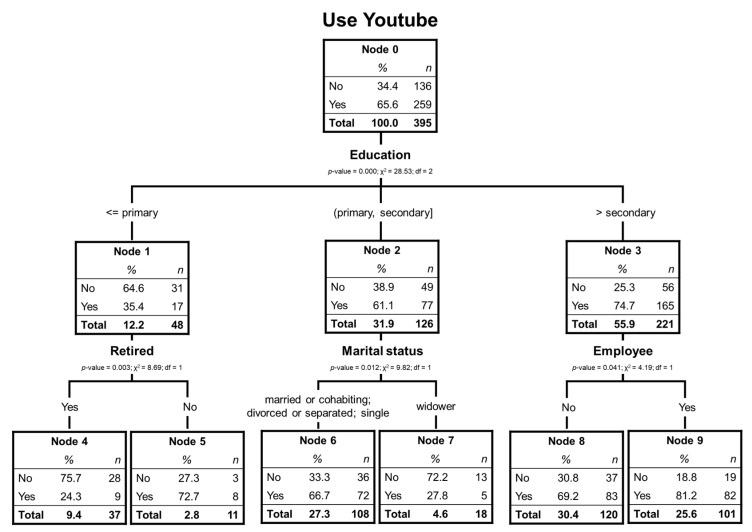
Decision tree of elderly YouTube users.

In this case, 65.6% of the respondents have used YouTube, and the independent variables that are significant to explain the different nodes are educational level, if he/she is retired, the marital status, and if he/she works. Figure 1 shows the tree obtained with a 2-level depth and 6 terminal nodes (which are the ones that no longer expand).

Figure 1 shows that the first most discriminating variable is the educational level, dividing the tree into three branches. Those with primary education have a much lower percentage of YouTube users (35.4%). In this branch, being retired or not is the next most significant variable, and it can be seen that node 4 is the one that includes the least YouTube users (24.3%). 

For those with medium studies, the next discriminant variable is marital status, indicating that those who are not widowed have a much higher percentage of use than widowers (66.7% vs. 27.8%). Among the respondents with higher education, the most discriminating variable is whether the respondent works. Node 9 has the highest proportion of elderly YouTube users (81.25%) who still work and have higher education.

Table 1 shows that the risk of misclassification is 27.6% (resubstitution method) or 29.6% (cross-validation method).

As can be seen in Table 2, 72.4% is well forecasted. Those who use YouTube are forecasted 94.6% while those who do not use it are not classified well (30.1%).

Table 3 shows that nodes 9, 5, and 8 have a percentage of YouTube users higher than usual (rates above 100%) and node six slightly above, indicating that they are the nodes with the most percentage of YouTube users among the total of the 6 terminal nodes.

### 4.2. Characterization of Elder Chileans Facebook Users

**Figure 2:**
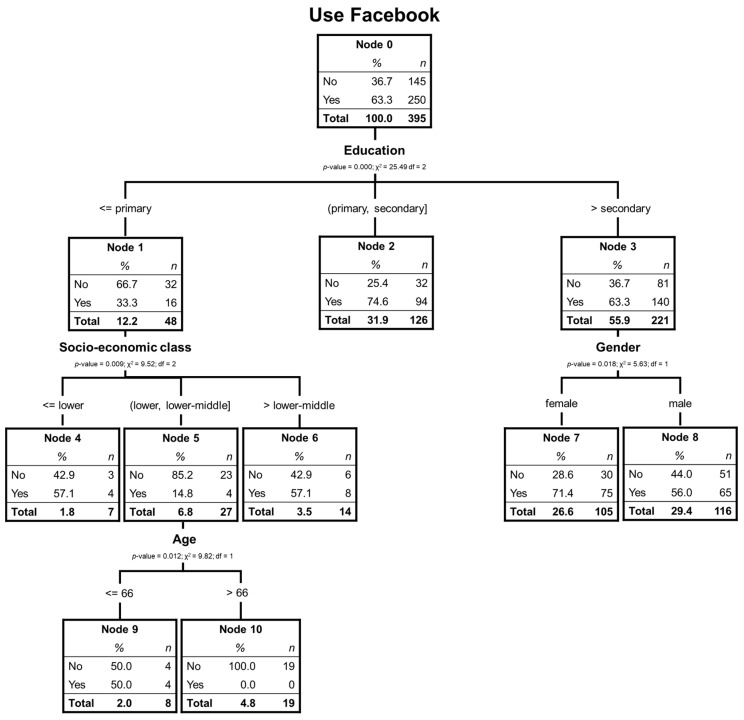
Decision tree of elderly Facebook users.

In this section, we analyze the elderly Chilean users of Facebook. The significant independent variables are educational level, socioeconomic class, age, and gender. A 3-level depth and 7 terminal nodes have been obtained. The corresponding tree is shown in Figure 2.

In the initial node, it can be seen that 63.3% of the respondents have used Facebook, being again the educational level the most relevant variable to classify. Node 2, which includes those with secondary studies, does not branch further and is the one with the highest percentage of users (74.6%). For those with higher education, the next crucial variable is gender, with women in this group having much higher use of Facebook than men (71.4% vs. 56.0%). Regarding those surveyed with primary studies, the next discriminant variable is socioeconomic class. Among those with a lower-middle-class, age is discriminatory, separating those over 66 years old, with 0% of users from the rest.

Table 4 shows that the risk of error in the classification is 31.9% and 34.7%, according to cross-validation method. Furthermore, in Table 5, we can see that 68.1% is well forecasted, especially those who use Facebook are classified very well (98.4%); those who do not use it are not classified well (15.9%).

Table 6 shows that nodes 2 and 7 are those with the most weight in Facebook use and those that best explain the respondents who use this social network the most.

### 4.3. Characterization of Elder Chileans Instagram Users

**Figure 3:**
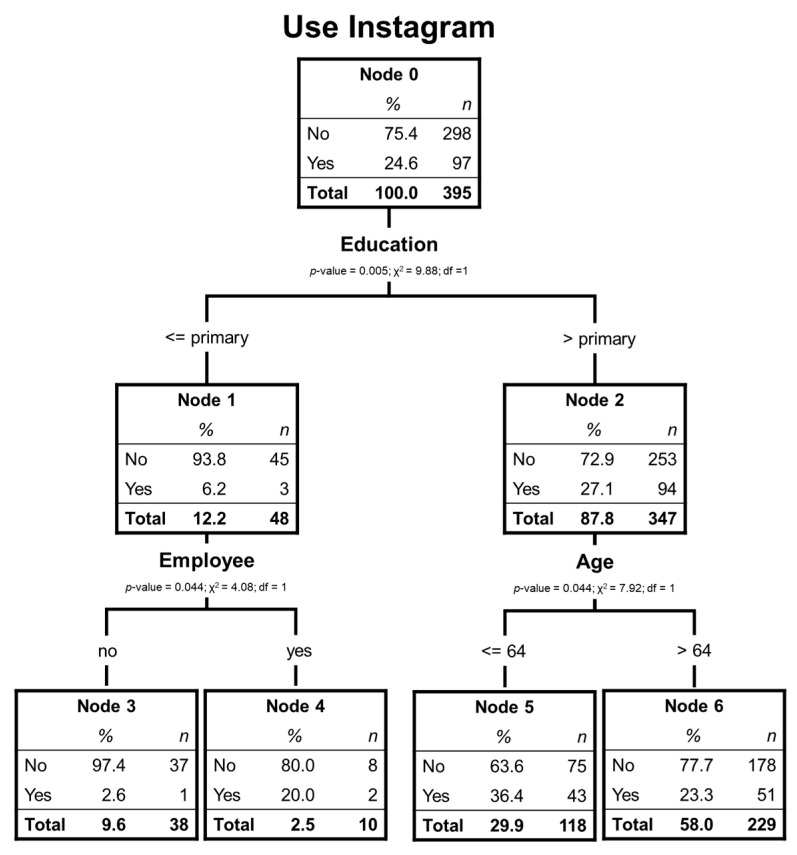
Decision tree of elderly Instagram users.

For this social network, the significantly relevant variables are educational level, whether the respondent works, and age. In this case, the tree has a 2-level depth and 4 terminal nodes (see Figure 3). Only 24.6% of respondents have used Instagram, which indicates that it is not one of the most used social networks by elder Chileans.

As in the case of YouTube and Facebook users, the educational level is the most relevant variable to classify. However, in this case, the algorithm only separates those who have primary studies from the rest. The elderly with primary studies are the vast majority of non-users (93.8%). In this branch, the next variable with incidence is if the respondent works. Among those who do work, the percentage of users who do not use Instagram is much lower than those of node 3 (80.0% vs. 97.4%). Among the respondents with secondary or higher studies with many more Instagram users than node 1, the most discriminant variable is age, which separates those over 64 years old. Taking into account that the youngest individuals are 60 years old, it indicates that the highest percentage of Instagrammers are in terminal node 5 (36.4%), who are people with secondary or higher education and between the ages of 60 and 64.

Table 7 shows that the risk of incorrect estimation of the model is 24.6%, the same value offered by cross-validation.

Table 8 shows that the model perfectly explains those who do not use Instagram, but it does not explain those who do use it and that these are few compared to the total.

Table 9 has been used as an objective explanation for those who do not use Instagram since this group is the one that has more reliability in the forecast. Node 3 is the one that most explains the profile of those who do not use Instagram.

### 4.4. Characterization of Elder Chileans Twitter Users

**Figure 4:**
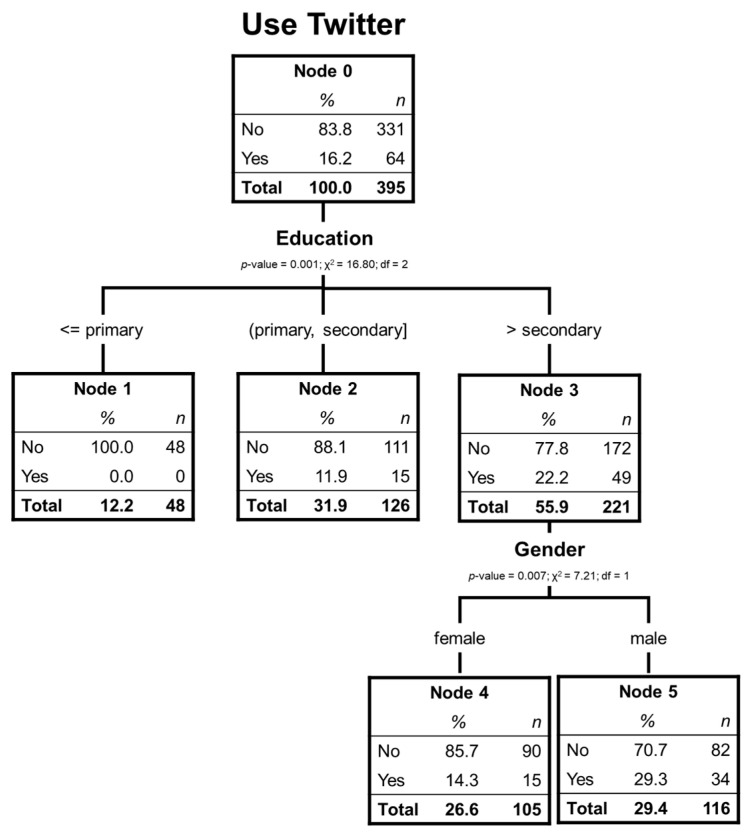
Decision tree of elderly Twitter users.

For this analysis, the significantly relevant variables are the educational level and gender (Figure 4). In this case, the tree has a 2-level depth and 4 terminal nodes. Only 16.2% of respondents have used Twitter which indicates that this social network is the one elder Chileans use the least.

As occurred previously, the educational level is the most relevant variable to classify; among those with primary studies, no one uses Twitter. Only one branch emerges among those with higher education, and it is the gender variable that discriminates significantly, with the percentage of male Twitter users being much higher than that of women in this group.

Table 10 shows that the risk of misclassification is shallow (16.2%), both by the replacement and cross-validation methods.

As in the previous case in which there are few users of this social network, it is correctly predicted not to use Twitter, but not to use it at all (see Table 11).

Table 12 shows that node 1 is the one that explains the most the non-use of Twitter, followed by node 2.

### 4.5. Characterization of Elder Chileans WhatsApp Users

**Figure 5:**
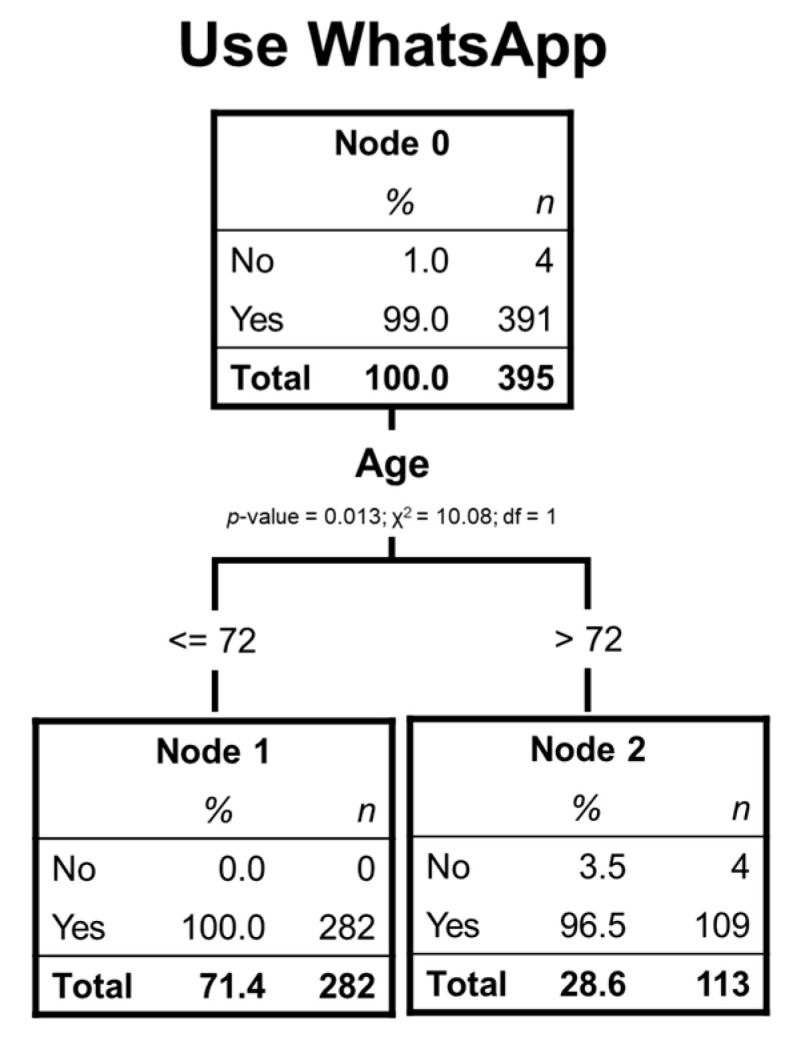
Decision tree of elderly WhatsApp users.

This case is particular since 99% of the respondents use this mobile application. Therefore, classification is severe; the only variable that discriminates significantly is age. The tree only has 2 terminal nodes and 1-level deep (see Figure 5).

In this particular case, 99% of respondents use this social network, age is the only significantly discriminating variable. This circumstance occurs when only four non-users are over 72 years old, separating nodes 1 and 2.

Table 13 shows that the risk of a substandard classification is almost nil since practically all the respondents use WhatsApp.

Table 14 shows that, as almost all respondents are users of this social network, its use is correctly predicted.

Table 15, in this case, has little meaning since the gain for nodes is less relevant when 99% are users of the social network.

Finally, Table 16 summarizes the results of the analyzes; in this table, the independent variables that classify the use of each SNS are detailed by levels.

## 5. Discussion

To our knowledge, very few studies have addressed the detection of segments with different behaviors in the use of SNS by older adults in Chile. The current research aims to contribute substantially to the knowledge about this phenomenon of the use of SNS by older people since it is not possible to understand this action except through the exploration of the heterogeneity of this population. Likewise, and given that the empirical analysis is conducted in Chile, the practical implications of these results will help both to establish differentiated lines of action in the field of public health for the promotion of the use of SNS in elderly adults, and to generate teaching methodologies in ICT adjusted to each detected segment.

First, it is possible to underline the fact that many differences appear among the various SNS both in their use and in their characterization. The results indicate that the WhatsApp communication application is used by 99% percent of elderly respondents, followed by YouTube (65.6%), Facebook (63.3%), Instagram (24.6%), being Twitter (16.2%) the one having the fewest users. This fact indicates that respondents widely use 3 of the top 5 SNS. Although the frequency of use of YouTube and Facebook is entirely consistent with international [38] and Chilean [39] statistics, the high use of WhatsApp is associated with a growing characteristic of the Chilean market. More than two-thirds of Chilean people said that they used WhatsApp at least once per hour during the day, and among these, baby boomer generation reports 74% the frequency of use of this application [39].

Another aspect to highlight is understanding which variables have more discriminating power in the characterization of the elderly users of these SNS. Undoubtedly, educational level is the most relevant of all; in general, it is observed that the more studies the person achieved, the more use is made of the different SNS analyzed in this study. Age is also significant in some subgroups obtained, and in general, it is observed that the older uses social networks less. This idea is also corroborated in some previous studies conducted in Chile [40]. Gender is also discriminant in some specific subgroups, women use less Twitter than men, and men use less Facebook than women. The literature could explain these differences. According to [41], females tend to exceed males in Facebook usage due to their stronger aspiration to maintain contact with friends and share photos. In contrast, Twitter demonstrates significant male bias around the world [42], Twitter’s users are more attentive to the social and political circumstances [43]. Lastly, it should be noted that whether the elderly are working has an impact on some segments; in general, those who have a job use more Instagram and YouTube.

The analysis carried out in this study allowed us to describe how older adults use each of the SNS studied. There are notorious differences between users of different platforms. Each SNS has its distinctive characteristics, which are better suited to some profiles than others. We will analyze each SNS next.

For YouTube, we have the most substantial users: the elderly with primary education and not retired (72.7%) and those with higher education and still working (81.2%). These results are well adjusted to understand YouTube as a tool for informal training [44]. The tutorial videos for carrying out different professional tasks fit well with the profile of identified users. On the other hand, we highlight the great ability of YouTube to integrate its contents in other networks, particularly to complement those others with greater importance of the written word, such as Twitter, Facebook, and WhatsApp.

Concerning Facebook, women with higher education (71.4%) stand out in the use of this social network concerning the rest of the segments found. In this segment, the level of users is much higher than in the other groups. These results that associate the use of Facebook with females are not surprising, if we consider the large number of studies that characterize females as more social than males [24,26]. In this sense, Facebook is one of the most popular SNS, with older users and a higher social focus, where users feel belonging to a community [45].

Regarding Instagram, data analysis allows us to identify non-users better. The non-users of Instagram are unusually high among people with primary studies who do not have a job (97.4% of non-users) and those with secondary or tertiary studies with more than 64 years old (77.7% of non-users). Currently, Instagram is an application widely used by younger generations, but little frequented by users over the age of 45. More than 65% of users are under 35 [46]. Given that Instagram is based on sharing photos and audiovisual content, we believe that it has high growth potential, especially among older adults with lower levels of study.

Analyzing the social network Twitter, we can also better identify non-users. A hundred percent of respondents with primary studies do not use this social network, 88.1% of those with secondary studies do not use it, and women with higher studies show 85.7% of non-use. Our results are consistent with other studies that have associated Twitter users with concrete segments and professions: journalists, politicians, musicians, and the media [43].

Regarding WhatsApp, the results show that 99% of the respondents are users, and older adults may not identify WhatsApp as an SNS, since it is less open than the rest. The great challenge may be creating tools specifically targeted for this application where older adults develop productive interactions. On the other hand, fake news is a threat shared by all SNS [47,48]; however, WhatsApp’s little open feature makes it especially sensitive to this risk. This threat can be a problem among older adults with a lower educational level.

If social networks were to be considered to disseminate public policy matters, such as messages related to health and well-being, the network that would allow reaching larger segments of older adults would be WhatsApp. The only discriminatory variable of this social network was age, with less use in people over 72 years old. Therefore, to take advantage of the extensive use of this social network, public policies should consider fomenting the use of Whatsapp in public circles. Besides, WhatsApp can be used to reach health services such as telemedicine to connect health providers with older adults.

The level of education strongly moderates other SNS. While Facebook and YouTube have better reach in segments of medium educational levels, Instagram and Twitter are used by better educated older people. Thus, to disseminate messages about public policies aimed at older people, another recommendation would be to use platforms such as Facebook or YouTube. It would be advisable to use short video format, animated and with messages in large or capital letters, which allow them to be shared by WhatsApp when the user considers that the content is worth sharing.

## 6. Conclusions

In summary, the results of this study suggest the existence of considerable differences between the various social networks analyzed in their use and characterization among elderly users. Educational level is the most discriminating variable, and gender influences the types of SNS use. 

Even though the study followed a rigorous research methodology, it is not without limitations: having surveyed only older adults who are users of social networks, collecting data from one country, and using only sociodemographic variables to segment elders. Future research could focus on comparing older users with non-users of these networks, extending the study to other countries and cultural fields, and using other psychographic and behavioral variables to segment elders.

To conclude, we emphasize three strengths of the research. First, the study addresses an important issue among older populations that have received little scholarly attention in non-western societies. Therefore, its findings are essential for understanding this phenomenon globally. Second, the study sample represents the heterogeneity of older adults in Chile, allowing the development of strategies at the country level to promote the use of SNS in this age segment. Third, the study provides a detailed methodology that can be used in other countries with a similar purpose.

## Figures and Tables

**Table 1:** Estimation risk for using YouTube (CHAID method).

Method	Estimation	Error Dev.
Resubstitution	0.276	0.022
Cross-Validation	0.296	0.023

**Table 2:** Classification success for using YouTube.

Observed	Predicted		
	No	Yes	Right%
No	41	95	30.1
Yes	14	245	94.6
Global%	13.9	86.1	72.4

**Table 3:** Earnings for nodes: target those who do use YouTube.

Node	Node		Gain		Answer%	Index%
	*N*	%	*N*	%		
9	101	25.6	82	31.7	81.2	123.8
5	11	2.8	8	3.1	72.7	110.9
8	120	30.4	83	32.0	69.2	105.5
6	108	27.3	72	27.8	66.7	101.7
7	18	4.6	5	1.9	27.8	42.4
4	37	9.4	9	3.5	24.3	37.1

**Table 4:** Estimation risk for using Facebook (CHAID method).

Method	Estimation	Error Dev.
Resubstitution	0.319	0.023
Cross-Validation	0.347	0.024

**Table 5:** Classification success for using Facebook.

Observed	Predicted		
	No	Yes	Right%
No	23	122	15.9
Yes	4	246	98.4
Global%	6.8	93.2	68.1

**Table 6:** Earnings for nodes: target those who do use Facebook.

Node	Node		Gain		Answer%	Index%
	*N*	%	*N*	%		
2	126	31.9	94	37.6	74.6	117.9
7	105	26.6	75	30.0	71.4	112.9
6	14	3.5	8	3.2	57.1	90.3
4	7	1.8	4	1.6	57.1	90.3
8	116	29.4	65	26.0	56.0	88.5
9	8	2.0	4	1.6	50.0	79.0
10	19	4.8	0	0.0	0.0	0.0

**Table 7:** Estimation risk for using Instagram (CHAID method).

Method	Estimation	Error Dev.
Resubstitution	0.246	0.022
Cross-Validation	0.246	0.022

**Table 8:** Classification success for using Instagram.

Observed	Predicted		
	No	Yes	Right%
No	298	0	100.0
Yes	97	0	0.0
Global%	100.0	0.0	75.4

**Table 9:** Earnings for nodes: target those who do use Instagram.

Node	Node		Gain		Answer%	Index%
	*N*	%	*N*	%		
3	38	9.6	37	12.4	97.4	129.1
4	10	2.5	8	2.7	80.0	106.0
6	229	58.0	178	59.7	77.7	103.0
5	118	29.9	75	25.2	63.6	84.2

**Table 10:** Estimation risk for using Twitter (CHAID method).

Method	Estimation	Error Dev.
Resubstitution	0.162	0.019
Cross-Validation	0.162	0.019

**Table 11:** Classification success for using Twitter.

Observed	Predicted		
	No	Yes	Right%
No	331	0	100.0
Yes	64	0	0.0
Global%	100.0	0.0	83.8

**Table 12:** Earnings for nodes: target those who do use Twitter.

Node	Node		Gain		Answer%	Index%
	*N*	%	*N*	%		
1	48	12.2	48	14.5	100.0	119.3
2	126	31.9	111	33.5	88.1	105.1
4	105	26.6	90	27.2	85.7	102.3
5	116	29.4	82	24.8	70.7	84.4

**Table 13:** Estimation risk for using WhatsApp (CHAID method).

Method	Estimation	Error Dev.
Resubstitution	0.010	0.005
Cross-Validation	0.010	0.005

**Table 14:** Classification success for using WhatsApp.

Observed	Predicted		
	No	Yes	Right%
No	0	4	0.0
Yes	0	391	100.0
Global%	0.0	100.0	99.0

**Table 15:** Earnings for nodes: target those who do use WhatsApp.

Node	Node		Gain		Answer%	Index%
	*N*	%	*N*	%		
1	282	71.4	282	72.1	100.0	101.0
2	113	28.6	109	27.9	96.5	97.4

**Table 16:** Independent variables by level in the decision trees that explain the use of SNS.

Level	YouTube	Facebook	Instagram	Twitter	WhatsApp
1	Education	Education	Education	Education	Age
2	Employee Retired Marital status	Gender Socio-economic class	Age Employee	Gender	
3		Age			
